# High-flow Oxygen and Nitric Oxide inhalation versus high-flow oxygen alone to prevent intubation in hypoxaemic Respiratory failure (HONOR): a pilot randomised controlled trial protocol

**DOI:** 10.1186/s40814-025-01726-1

**Published:** 2025-11-26

**Authors:** Luke Churchill, Oystein Tronstad, Karen Hay, Peter J. Thomas, Kiran Shekar

**Affiliations:** 1 Physiotherapy Department, The Prince Charles Hospital, Chermside, Brisbane, QLD 4032 Australia; 2 Critical Care Research Group, The Prince Charles Hospital, Brisbane, Australia; 3 QIMR Berghofer Medical Research Institute, Brisbane, Australia; 4 Department of Physiotherapy, Royal Brisbane and Women’s Hospital, Brisbane, Australia; 5 Department of Intensive Care, Royal Brisbane and Women’s Hospital, Brisbane, Australia; 6 Adult Intensive Care Services, The Prince Charles Hospital, Brisbane, Australia; 7 School of Medicine, The University of Queensland, Brisbane, Australia

**Keywords:** Acute respiratory failure, Endotracheal intubation, Invasive mechanical ventilation, Inhaled nitric oxide, High-flow oxygen, Oxygen therapy

## Abstract

**Background:**

When conventional oxygen therapies fail, endotracheal intubation and invasive mechanical ventilation are the current standard of care in patients with acute hypoxaemic respiratory failure. However, invasive mechanical ventilation is associated with increased hospital and intensive care length of stay, healthcare costs, and morbidity and mortality. Inhaled nitric oxide has the potential to treat hypoxaemia and potentially prevent the need for invasive mechanical ventilation.

**Aims and objectives:**

The objective of this study is to examine the feasibility and effectiveness of high-flow oxygen and nitric oxide gas inhalation compared with high-flow oxygen alone in preventing invasive mechanical ventilation for patients with acute hypoxaemic respiratory failure.

**Methods:**

In this pilot, randomised controlled feasibility study, 40 patients admitted to the intensive care unit with acute hypoxaemic respiratory failure will be randomised on a 1:1 ratio to receive one of two interventions: high-flow oxygen and nitric oxide gas inhalation (intervention) or high-flow oxygen alone (control) via high-flow nasal cannula. Feasibility, demographic, outcome, and safety data will be collected at several timepoints during participants’ admission.

**Discussion:**

This protocol outlines a structured method for investigating the effects of inhaled nitric oxide gas in preventing invasive mechanical ventilation for patients with acute hypoxaemic respiratory failure. Considering the risks, costs, and poorer outcomes associated with invasive mechanical ventilation, less invasive means of respiratory support warrant further investigation. The study will assist in planning a larger, multi-centre definitive trial.

**Trial registration:**

This study is registered on the Australian New Zealand Clinical Trials Registry (ANZCTR): ACTRN12622001411730. Registered 4th September 2022, https://www.anzctr.org.au/Trial/Registration/TrialReview.aspx?id=384881.

**Supplementary Information:**

The online version contains supplementary material available at 10.1186/s40814-025-01726-1.

## Introduction

Acute hypoxaemic respiratory failure is a common and life-threatening consequence of a diverse group of conditions [1, 2]. When conventional oxygen therapies (COT) (≤ 15 L/min oxygen via nasal prongs, cannula or mask) [3] or non-invasive ventilation (NIV) fail to correct hypoxaemia, invasive mechanical ventilation (IMV) is required. The use of IMV is common throughout the world and is increasing annually [4, 5]. Patients on IMV represent approximately 3% of acute hospitalisations and 30% of intensive care unit (ICU) admissions both internationally and in Australia [1, 2, 6–8]. However, outcomes of IMV are highly dependent on factors such as aetiology, age, co-morbidities, and severity of illness [9].

Whilst often a life-saving intervention, intubation and IMV are not without inherent risks. Risks include laryngeal injury, injury to lung parenchyma, adverse haemodynamic consequences (e.g. decreased venous return, blood pressure, and cardiac output) and predisposition to infection (e.g. ventilator-associated pneumonia (VAP)) [10–13]. Emergent endotracheal intubation also carries an especially high risk, with reported intubation-related cardiac arrest (occurring within 20 min after successful intubation) rates of up to 23% [14]. Despite evidence demonstrating a decrease in mortality rates over time, up to 30–40% of patients receiving IMV will not survive their ICU admission [15] and many survivors experience reduced quality of life, impaired physical function, and increased psychological conditions such as depression, anxiety and post-traumatic stress disorder [16, 17]. Invasive mechanical ventilation is also associated with significant increases in hospital and ICU length of stay (LOS) [6, 18], with increased cost burden ranging from 25 to 59% extra per ICU patient per day receiving IMV [7, 19, 20].

Reducing the incidence, risks, and costs associated with IMV is a major priority for healthcare providers, consumers, health system administrators, taxpayers, and policymakers [6]. By averting an artificial airway and IMV, patients with acute respiratory failure (ARF) supported with less invasive means can often avoid intravenous sedation and costly complications, such as VAP, ICU-acquired weakness, and line sepsis [6]. Avoiding intubation also facilitates patient-centric aims of early rehabilitation, speech, and oral feeding which may improve patient outcomes and reduce hospital and ICU LOS.

High-flow oxygen (HFO_2_) delivered through nasal cannula and NIV are routinely used in the treatment of ARF [21–23]. In a randomised clinical trial in patients with ARF, HFO_2_ therapy demonstrated a non-significant reduction in IMV compared with COT and NIV, however, resulted in a better 90-day survival [24]. In this study, the rates of intubation were lowest in the HFO_2_ therapy group (38%), compared with COT and NIV (47% and 50% respectively). The leading cause of intubation across groups (> 70%) was worsening ARF and hypoxaemia, warranting further investigation into the most optimal strategies to mitigate this.

The addition of inhaled Nitric Oxide (iNO) to nasal HFO_2_ may allow hypoxaemia to be corrected and potentially avoid IMV [25, 26]. As a potent vasodilator, iNO has the ability to provide selective pulmonary vascular dilation in well-ventilated sections of the lungs, improving ventilation-perfusion mismatch [26, 27]. Significant improvements in oxygenation have been demonstrated in infants with ARF on nasal continuous positive airway pressure with iNO [28]. Results within the adult population remain inconsistent, with short-term improvements in the ratio of the partial pressure of arterial oxygen (PaO_2_) to fraction of inspired oxygen (FiO_2_) ratio (PF) often being transient or not sustained [29–33]. However, the primary means of delivering iNO was either via COT or mechanical ventilation and not via nasal HFO_2_.

Literature demonstrating the effects of iNO combined with HFO_2_ (HFO_2_+iNO) in the adult population remains sparse. One multi-centre cohort study evaluated the effectiveness of HFO_2_+iNO in patients with respiratory failure from coronavirus disease (COVID-19) [34]. In this population, HFO_2_+iNO did not reduce oxygen requirements in the majority of patients. However, a subset of patients considered responders (defined as a decrease in supplemental oxygen delivered via high-flow nasal cannula (HFNC) 12 h after iNO initiation) had a trend toward decreasing need for IMV compared to non-responders [34]. Outside of this patient population, only case reports exist of the successful use of HFO_2_+iNO for preventing IMV. These reports demonstrated improvements in oxygenation within the hospital setting and maintaining safe oxygen levels during transport to and from hospital [35, 36]. Therefore, to further investigate the potential benefits of HFO_2_+iNO in preventing IMV, further research is needed in its potential to reduce the need for IMV.

## Aims

The primary aim of this pilot study is to examine the feasibility of comparing HFO_2_+iNO gas inhalation to HFO_2_ alone in patients with hypoxaemic, non-hypercapnic ARF. Several clinical outcomes (including safety and intubation rates) will also be examined. Based on the physiologic rationale [25, 37], prior investigations [28, 35, 36], and our anecdotal experience, we hypothesise that delivery of HFO_2_+iNO therapy is feasible, safe, and superior to HFO_2_ alone in preventing IMV in patients with ARF. This pilot study is a requisite initial step in exploring the proposed intervention [38] in preparation for a larger scale, multi-centre definitive trial. The feasibility and safety outcomes of the study protocol will inform future budget and protocol development whilst providing initial effect estimates to inform sample size calculation.

## Methods

### Design

**Fig. 1:**
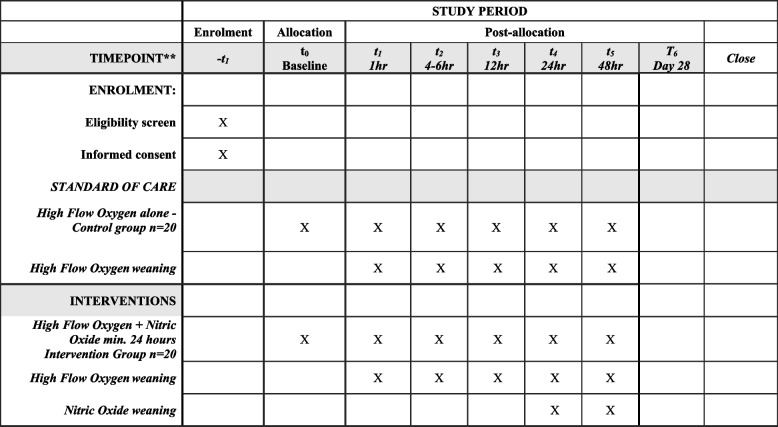
Schedule of enrolment and interventions

**Fig. 2:**
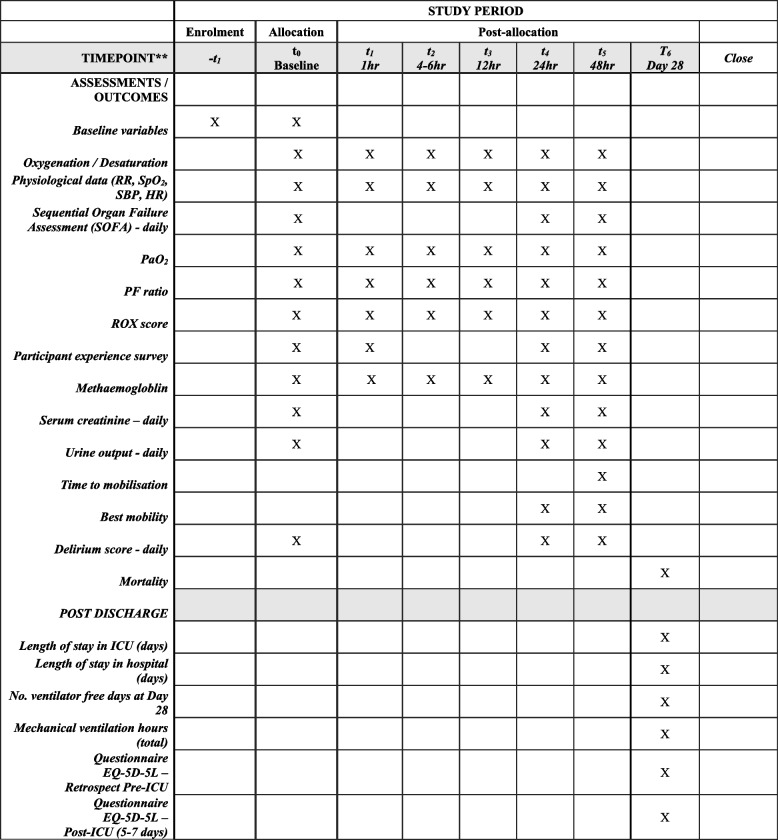
Schedule of assessments and collection of outcomes

This will be an open label, 1:1 parallel group, single-centre, pilot (*n* = 40) randomised controlled trial. The study protocol has been reported using the Standard Protocol Items: Recommendations for Interventional Trials (SPIRIT) statement guidelines [39], and the associated publication from the study will be reported in accordance with the CONSORT extension for randomised pilot and feasibility trials [40]. The SPIRIT figures (Figs. 1 and 2) outline the schedule of enrolment, interventions, and assessments [39].

### Setting

This study will be conducted in a 27-bed adult ICU at a large urban Australian tertiary referral hospital specialising in cardiothoracic surgery and medicine.

### Sample size

As a pilot trial, it is not required to be powered to detect statistical significance. A sample of 40 eligible patients will be recruited over a period of 12–18 months. Assuming the proportion of patients who progress to IMV is 0.38 [24], a sample of 20 per group will produce an exact 95% CI width of 0.44.

### Recruitment and consent

Patients presenting with type I acute hypoxaemic respiratory failure (PaO_2_ < 60 mm Hg with normal or subnormal PaCO_2_) [41] will have arterial blood gas (ABG) sampling as per standard care for this patient cohort. Aligning with previous research investigating HFO_2_ in ARF [24], if the PF ratio is less than 300 mm Hg, they will be screened for suitability to participate in the study by a member of the research team. Written informed consent will be obtained from all suitable patients, their next of kin, or another substitute decision maker (SDM) as appropriate. Owing to the nature of their injury, potential participants are unlikely to have the capacity to provide written consent at the time of recruitment. Verbal consent from an SDM will be allowed if geographical or infection control visitor restrictions prevent face-to-face informed consent processes. Written consent will be sought later if a SDM is able to attend in person or are able to return a consent form provided to them via email. Should there be no SDM available initially, study procedures will be performed and consent to continue will be sought from the SDM, or the participant (should they regain capacity to consent) within a maximum of three days. Participants and/or their SDM can decline study assessments and discontinue participation at any time without explanation or penalty.

Simple random allocation will be applied, with participants allocated in a 1:1 ratio to either the control or intervention arm via a computer-generated random sequence program.

### Inclusion and exclusion criteria

Patients will need to meet all inclusion criteria and no exclusion criteria (Table 1) to be eligible to participate.

**Table 1:** Inclusion/exclusion criteria for review

Inclusion	Exclusion
• Age ≥ 18 years • Admitted to ICU • De novo type I respiratory failure (hypoxaemia in the absence of chronic lung condition) [24] • PF ratio < 300 mm Hg • High-flow nasal cannula determined by ICU medical team to be primary method for delivery of oxygen therapy • Anticipated HFO_2_ requirement > 24 h • Arterial line in-situ for blood gas sampling • Ability to provide informed consent, or consent via a SDM	• Congenital or acquired methaemoglobinaemia reductase deficiency • Bleeding diathesis • Intracranial haemorrhage • Severe left ventricular failure • Underlying chronic respiratory failure or exacerbation of asthma (including chronic obstructive pulmonary disease (COPD) or other chronic respiratory disease) • Documented cardiogenic pulmonary oedema or acute coronary syndrome • Hypercapnic respiratory failure with PaCO_2_ > 45 mm Hg • Deterioration of neurologic status demonstrated by Glasgow Coma Scale (GCS) ≤ 12 • Urgent need for intubation (evaluated by the medical officer in charge) • Haemodynamic instability (defined by systolic arterial blood pressure < 90 mm Hg or mean arterial blood pressure < 65 mm Hg) • Use of vasopressors • Do not intubate orders • Enrolled in any other trial of targeted oxygen therapy

*ICU* intensive care unit, *HFO*_*2*_ high-flow oxygen, *Hg* mercury, *mm* millimetres, *PaCO*_*2*_ partial pressure of arterial carbon dioxide, *PF* ratio of partial pressure of arterial oxygen to fraction of inspired oxygen, *SDM* substitute decision maker

### Blinding

Due to the nature of the interventions, participants receiving the interventions and the clinicians providing care will be aware of the treatment allocation. During the consent process, participants will be informed of the two interventions and be made aware that both are considered standard of care at the study site.

### Study interventions

Participants will receive one of two interventions: HFO_2_ alone or HFO_2_+iNO as detailed below.

High-flow oxygen (HFO2) alone (control group):

- Initial FiO_2_ set at 100% with an initial flow of 60 L/min
- FiO_2_ will be titrated down in the first hour, targeting oxygen saturation (SpO_2_) > 92% and PaO_2_ > 60 mm Hg
- Further reduction in FiO_2_ every subsequent two hours maintaining SpO_2_ > 92% and PaO_2_ > 60 mm Hg
- Flows will be reduced in increments of 10 L/min if needed for tolerance, to a minimum of 30 L/min
- Heated humidification to ensure delivery of HFO_2_ into the nares at a temperature set at 37 °C

High-flow oxygen and nitric oxide (HFO_2_+iNO) (intervention group):

High-flow oxygen therapy as described above, with the addition of:

- iNO initially set at 20 parts per million (ppm) via HFNC by medical team, administered by ICU nursing staff, and weaned as per the following:
  ◦ After 24 h of study drug administration, wean iNO 1 ppm every 20 min or as directed by the treating ICU Consultant, provided:
    ▪ PaO_2_ > 60 mm Hg and SpO_2_ > 92% for greater than 6 h with an FiO_2_ ≤ 50%
      FiO_2_ may be increased up to a maximum of 60% to compensate for any drop in oxygenation
  ◦ If nil decrement in oxygenation seen, iNO will be further weaned at a rate of 1 ppm/20 min to 0 ppm) or as directed by ICU Consultant
  ◦ If desaturation persists (SpO_2_ < 92%) for > 15 min, iNO will be returned to the most recent level prior to weaning or escalated up to a maximum of 20 ppm to maintain patient oxygenation
  ◦ iNO inhalation will be maintained if the patient in the HFO_2_ + iNO arm requires NIV

### Blood sampling

As per standard of care for patients with ARF within ICU, regular ABGs (approximately four to six per day) are collected via an arterial line to monitor several indices within the blood (such as PaO_2_, PaCO_2_, PF ratios, serum creatinine, and methaemoglobin (MetHb) levels). Further ABGs can also be requested by the medical treating team as clinically indicated. To monitor responses to therapy within the study, participants will have several ABGs completed at pre-determined timepoints (Fig. 1), most of which will occur as part of standard care within ICU. However, specific timepoints (such as six and 12 hours after study inclusion) may require additional ABGs. In these instances, approximately 4 ml of blood will be collected from participants via their arterial line for analysis within ICU. No further ABGs will be collected if/when an arterial line is removed, therefore, negating the requirement of an arterial stab.

### Intervention timing

Timing of interventions and assessments during the study will occur via the schedules outlined in Figs. 1 and 2.

### Outcome measurements

Primary outcome.

- Feasibility
  ◦ Recruitment (at least 75% of all eligible patients recruited using approved consent methods)
  ◦ Retention (at least 80% of consented pts remaining within study)
  ◦ Protocol fidelity (at least 70% of pts in intervention group receiving HFO_2_+iNO for at least 22 h a day (accounting for times where/if iNO is ceased (e.g. transport, investigations, mobility away from the bedspace))
  ◦ Data collection procedures (at least 90% of in-person data collected at each study time point)
- Clinical outcomes (specific timepoints in Fig. 2)
- Number of patients in each arm progressed to IMV within 28 days
- MetHb levels measured via ABGs
- Daily serum creatinine and urine output levels to monitor renal function and/or renal impairment
- Change in PaO_2_ relative to baseline
- Change in PF ratio relative to baseline
- Physiological data
  ◦ Respiratory rate (RR)
  ◦ SpO_2_
  ◦ Systolic blood pressure (SBP)
  ◦ Heart rate (HR)
- Illness severity as estimated by daily sequential organ failure assessment (SOFA) scores [42]
- Worst daily PF ratio
- ROX index scores
- Reason for intubation
  ◦ Respiratory failure
  ◦ Circulatory failure
  ◦ Neurological failure
  ◦ Surgery
- Participant experience survey (Appendix 1)
- ICU LOS
- Hospital LOS
- Mechanical ventilation hours
- Number of ventilator free days at day 28
- Time to mobilisation (minimum classification 4 (standing) on the ICU mobility scale) [43]
- Best mobility (within first 24 h and during whole length of stay)
- Daily delirium incidence (Confusion Assessment Method for the ICU (CAM-ICU) score)
- Retrospective pre-ICU EuroQol 5-Dimension 5-Level (EQ-5D-5L) questionnaire
- EQ-5D-5L (collected within 5–7 days of discharge from the ICU)
- Need for anxiolytic and sedative medication during admission
- ICU mortality

### Data collection

Eligibility and recruitment data will be gained from the electronic screening log. The screening log will be used to document all patients who have been screened and identify those who consented to enrol in the study and those who were not enrolled, remarking the specific reason for exclusion. Data in relation to demography, physiology, admission severity of illness, haemodynamic data, details of haemodynamic support, and hepatic and renal functions will be collected by local data managers from electronic medical records.

Safety data will be collected for each patient including (i) MetHb levels, (ii) daily serum creatinine and urine output levels to monitor renal function and/or renal impairment, and (iii) adverse and serious adverse events (see section "Data handling and record keeping" below for further details).

### Data handling and record keeping

Case Report Forms (CRF’s) will be collected initially in hard copy before being transferred to an electronic format which will be stored on a password protected Queensland Health server. Paper documents will be stored in a secured locked cabinet at the study site under the supervision of the principal investigator and study coordinator. An electronic copy of the data will be maintained via a password protected spreadsheet, and the principal investigator and study coordinator will be responsible for its management.

The study team will ensure all steps are taken to maintain confidentiality and security over the study documentation. Documents will be maintained for a minimum of 15 years following the completion of the study, as per Good Clinical Practice (GCP) guidelines.

### Safety considerations

#### Methaemoglobin levels across groups

Nitric oxide oxidises heme iron to the ferric state, resulting in the formation of MetHb [44]. Methaemoglobin has higher oxygen affinity and decreased oxygen-carrying capacity due to fewer hemes to bind oxygen [45]. Safe levels of MetHb within previous trials for iNO are considered < 5% [27]. Pooled data (1275 participants with ARDS receiving iNO) demonstrated MetHb levels > 5% for four participants in the iNO group and three participants in the control group [33]. Whilst this risk remains extremely low, MetHb levels will be monitored in all participants in this study via ABGs during the timepoints listed in Fig. 2.

#### Renal impairments across groups

Previous literature has demonstrated that iNO may induce renal impairment [33]. Depending on the definition, renal impairment in participants receiving iNO range from 5 to 13% [29, 46, 47]. However, of these results, only one study demonstrated a statistically significant increase in renal impairment in the intervention group when comparing to a control group of COT [47]. To further examine rates of renal impairment within this study, daily serum creatinine and urine output levels will be collected and compared across both groups. Renal impairment will be classified by the Acute Kidney Injury Network (AKIN) criteria [48] (Appendix 2).

#### Adverse and serious events

Any adverse event (AE) associated with the conduct of the study will be collected and reported to the study sponsor (Metro North Hospital and Health Service). Additionally, an annual safety report will be provided to the approving human research ethics committee in line with the local requirement at the study site.

The defined AEs for the trial are:

- Renal complications (including new haemofiltration/dialysis and/or acute kidney injury defined by the AKIN classification/staging system of acute kidney injury, Appendix 2)
- MetHb levels > 5%

Serious adverse events (SAEs):

The definition of a SAE is one that fulfills at least one of the following:

- Is fatal—results in death
- Is life threatening
- Requires prolongation of existing hospitalisation
- Results in persistent or significant disability or incapacity [49]

Given that critically ill patients are likely to meet any of the above listed criteria during their ICU admission, only SAE’s that are thought to be related to the study will be reported. Serious adverse events will be reported to the study sponsor within 24 h of becoming aware of the event. For all events, a medically qualified study investigator will review the event and assign the causality relationship between the study intervention and the event (possibly, probably, or definitely related). Serious adverse events will also be periodically reviewed by an independent Data and Safety Monitoring Committee (DSMC). The DSMC consists of suitably qualified experts, including medical specialists and a biostatistician. There are no competing interests from the DSMC, and they remain independent from the study sponsor. A DSMC charter is kept within the study site file, only accessible to study personnel. Interim analysis of key efficacy and safety data will be undertaken by the DSMC when 50% of the projected total number of participants have been evaluated for the primary endpoint. The DSMC will meet after 25%, 50%, and 100% of the projected total number of participants have been recruited. After each meeting the DSMC will make one of several recommendations that may include continuing the study, proposing protocol changes, extending recruitment, or stopping the study early. If protocol changes are required, the trial registry will be updated accordingly, and study personnel will be notified and provided with the new protocol.

### Statistical analysis

The criterion for success of the pilot study is that the definitive trial will be feasible if fidelity to the protocol is at least 70%. We expect protocol fidelity to be ~ 85%. With a sample size of 40, the estimated fidelity of 85% can be estimated with a 95% confidence interval of width 22% (74%–96%). The lower bound of this 95% confidence interval is above the specified level to claim success. If fidelity is less than that specified, reasons will be investigated, and the protocol modified accordingly.

Consistent with published recommendations for pilot studies, we will refrain from a detailed inferential statistical analysis in this pilot study [38]. The sample size is based on the pragmatics of recruitment and the necessities for examining feasibility. Inclusion of HFO_2_ as control group in this pilot is deliberate and will allow realistic examination of recruitment, randomisation, and implementation of interventions.

Categorical variables will be summarised as frequencies (percentage) and continuous measures will be summarised as mean (standard deviation) or median (interquartile range) as appropriate. Binary primary outcome measures for each group will be presented with estimated proportions with 95% confidence intervals to convey precision. Kaplan-Meier curves will be plotted to explore time from enrolment to intubation or death in each group. For outcome measures with continuous repeated measures, within-group change, between-group differences, and differences in rates of change over time will be explored using mixed effects linear regression modelling.

## Discussion

The use of IMV is increasing annually [4, 5] but carries several risks to patients including VAP, adverse haemodynamic responses, and potential injuries to the upper airway and lung parenchyma [10–13]. Furthermore, significant increases in hospital and ICU LOS, and healthcare costs have been demonstrated for patients receiving IMV compared to non-ventilated patients [6, 7, 18–20]. Whilst the addition of iNO has demonstrated positive trends for reducing the need for IMV in certain patient populations [34–36], these results remain inconsistent.

The use of iNO with HFO_2_ offers a less invasive form of respiratory support. Reducing the incidence of IMV and IMV-related complications will improve the patient experience, patient outcomes, reduce ICU and hospital LOS [6], and result in substantial cost saving for the health service. Without IMV, patients need not go into induced coma, can retain autonomy, eat, drink, exercise, and rehabilitate which may translate into better long-term physical, cognitive, and psychological outcomes. Equally, integrating patients’ feedback and self-reported experiences across groups is important to achieve appropriate health care decisions that integrate both health care staff and patients.

## Limitations

This single-centre, pilot study will sample 40 patients. As a pilot study, it is not powered to detect statistically significant differences in primary and/or secondary outcomes between groups. The ICU undertaking the study specialises in cardiothoracic medicine and surgery. Therefore, the results of the study may not be generalisable to other patient cohorts. With several consecutive timepoints allocated for blood collections to assess oxygenation and safety measures (Fig. 2), adherence in obtaining these measures may fluctuate, dependent on patient acuity. Finally, obtaining participant experience surveys may at times prove difficult, depending on factors like delirium and the level of respiratory distress a patient experiences at this timepoint.

## Trial status

As at the time of writing this manuscript, the study has not commenced recruitment. The study protocol (version 2, 13th December 2024) has been approved by an authorised human research ethics committee (HREC/2024/MNHA/112848). Study recruitment is planned to commence June 2025 and be completed by December 2026.

## Supplementary Information

Supplementary Material 1: Appendix 1. Participant Experience Survey [24, 50, 51]. Appendix 2. The AKIN classification/staging system of acute kidney injury [48].

## Data Availability

Not applicable.

## Abbreviations

ABG: Arterial blood gas
AKIN: Acute Kidney Injury Network
AE: Adverse event
ARF: Acute respiratory failure
CAM-ICU: Confusion Assessment Method for the ICU
CRF: Case Report Form
COT: Conventional Oxygen Therapies
COVID-19: Coronavirus disease
EQ-5D-5L: EuroQol 5-Dimension 5-Level
FiO_2_: Fraction of inspired oxygen
GCP: Good clinical practice
GCS: Glasgow Coma Scale
HFNC: High-flow nasal cannula
HFO_2_: High-flow oxygen
HR: Heart rate
ICU: Intensive care unit
IMV: Invasive mechanical ventilation
iNO: Inhaled nitric oxide
LOS: Length of stay
MetHb: Methaemoglobin
NIV: Non-invasive ventilation
PaCO_2_: Partial pressure of arterial carbon dioxide
PaO_2_: Partial pressure of arterial oxygen
PF: Ratio of PaO_2_ to fraction of inspired oxygen
PPM: Parts per million
ROX: Respiratory rate-oxygenation
RR: Respiratory rate
SAE: Serious adverse event
SBP: Systolic blood pressure
SDM: Substitute decision maker
SOFA: Sequential Organ Failure Assessment
SpO_2_: Pulse oximetry
VAP: Ventilator-associated pneumonia
