# Supplementary material for: High-flow Oxygen and Nitric Oxide inhalation versus high-flow oxygen alone to prevent intubation in hypoxaemic Respiratory failure (HONOR): a pilot randomised controlled trial protocol
Source: Pilot Feasibility Stud. 2025 Nov 26;11:149. doi: 10.1186/s40814-025-01726-1 (PMC12659384; doi:10.1186/s40814-025-01726-1)
Supplement: Supplementary file 1 — Supplementary Material 1: Appendix 1. Participant Experience Survey [24, 50, 51]. Appendix 2. The AKIN classification/staging system of acute kidney injury [48]. [file 40814_2025_1726_MOESM1_ESM.docx]

**APPENDICES**

**Appendix 1**

Participant Experience Survey.

Researcher: Participant ID:

**Baseline (T0):**

**Q1**. Please rate your current breathlessness using the scale^50^ below:


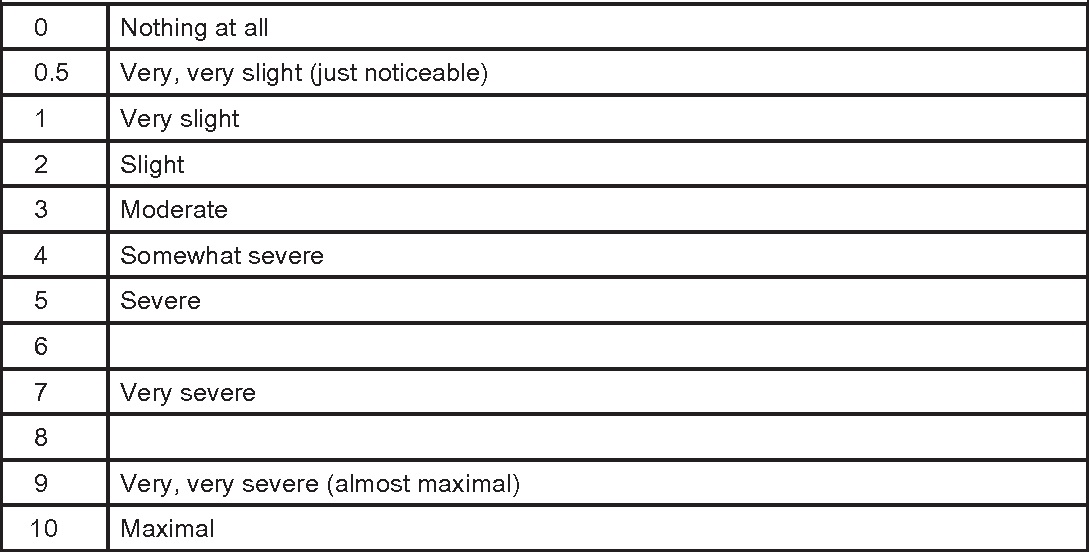


Answer:…………………………………………

**Q2**. Please rate your current breathing discomfort with the scale^51^ below (0 = no discomfort, 10 = maximal imaginable discomfort)?

0 1 2 3 4 5 6 7 8 9 10

**Q3**. Please rate your current level of anxiety using the scale^52^ below.

| Not at all anxious  (1) | A little anxious  (2) | Moderately anxious (3) | Very anxious  (4) | Extremely anxious  (5) |
| --- | --- | --- | --- | --- |
|  |  |  |  |  |

**1 hour (T1):**

**Q1**. Please rate your current breathlessness using the scale^50^ below:


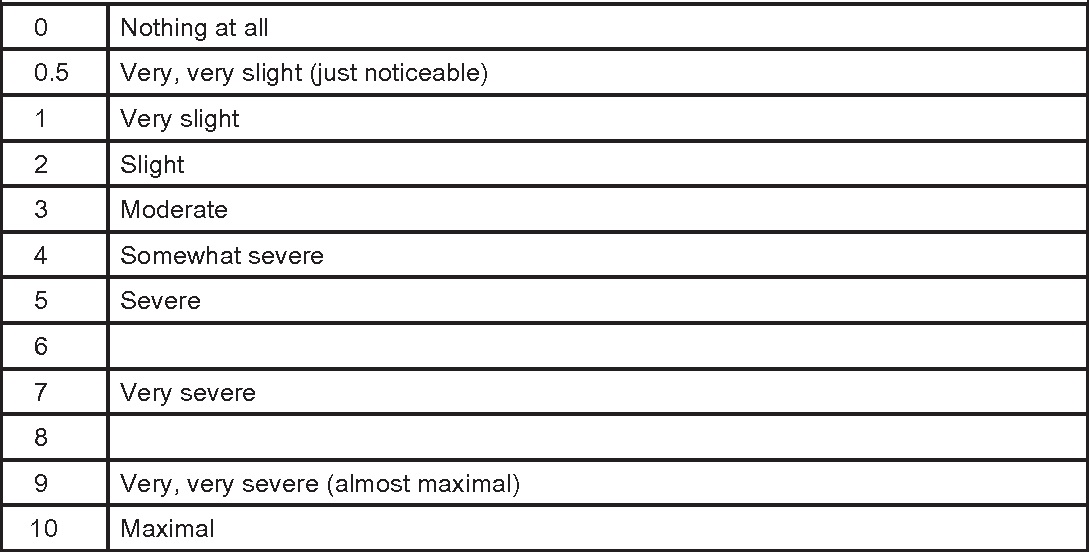


Answer:…………………………………………

**Q2**. Please rate your current breathing discomfort with the scale^51^ below (0 = no discomfort, 10 = maximal imaginable discomfort)?

0 1 2 3 4 5 6 7 8 9 10

**Q3**. Using the scale^51^ below, please rate any changes in your comfort levels with breathing since commencing the oxygen or nitric oxide gas:

| Marked deterioration  (-2) | Slight deterioration  (-1) | No change (0) | Slight improvement  (+1) | Marked improvement  (+2) |  |
| --- | --- | --- | --- | --- | --- |
|  |  |  |  |  | |

**Q4**. Please rate your current level of anxiety using the scale^52^ below.

| Not at all anxious  (1) | A little anxious  (2) | Moderately anxious (3) | Very anxious  (4) | Extremely anxious  (5) |  |
| --- | --- | --- | --- | --- | --- |
|  |  |  |  |  | |

**24 hours (T4):**

**Q1**. Please rate your current breathlessness using the scale^50^ below:


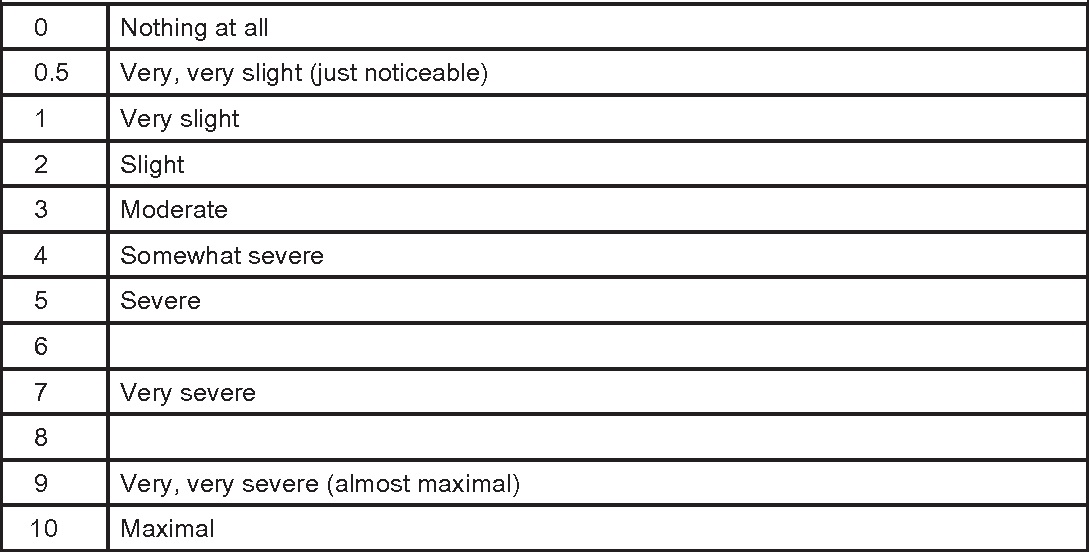


Answer:…………………………………………

**Q2**. Please rate your current breathing discomfort with the scale^51^ below (0 = no discomfort, 10 = maximal imaginable discomfort)?

0 1 2 3 4 5 6 7 8 9 10

**Q3**. Using the scale^51^ below, please rate any changes in your comfort levels with breathing since commencing the oxygen or nitric oxide gas:

| Marked deterioration  (-2) | Slight deterioration  (-1) | No change (0) | Slight improvement  (+1) | Marked improvement  (+2) |  |
| --- | --- | --- | --- | --- | --- |
|  |  |  |  |  | |

**Q4**. Please rate your current level of anxiety using the scale^52^ below.

| Not at all anxious  (1) | A little anxious  (2) | Moderately anxious (3) | Very anxious  (4) | Extremely anxious  (5) |  |
| --- | --- | --- | --- | --- | --- |
|  |  |  |  |  | |

**48 hours (T5):**

**Q1**. Please rate your current breathlessness using the scale^50^ below:


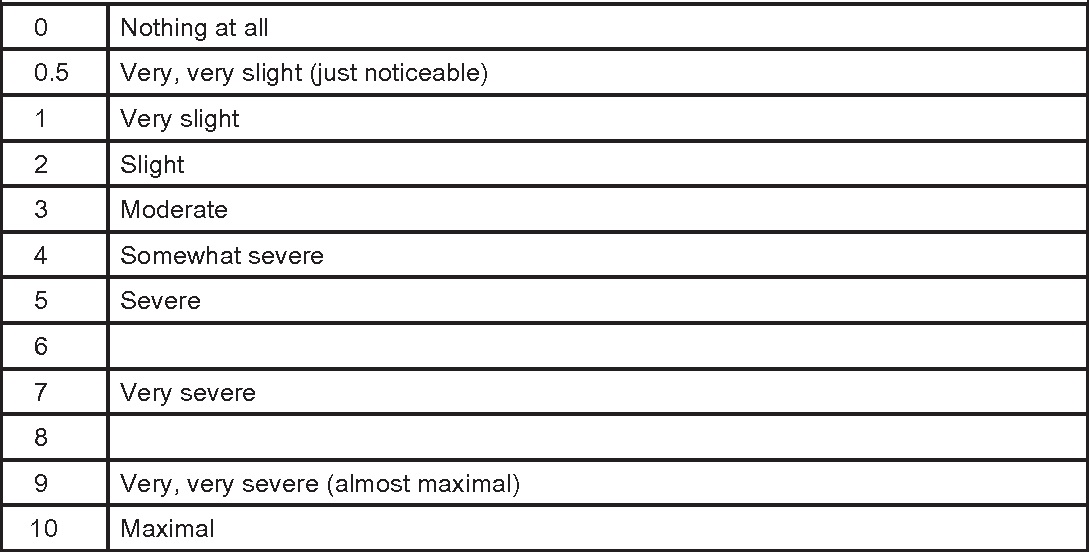


Answer:…………………………………………

**Q2**. Please rate your current breathing discomfort with the scale^51^ below (0 = no discomfort, 10 = maximal imaginable discomfort)?

0 1 2 3 4 5 6 7 8 9 10

**Q3**. Using the scale^51^ below, please rate any changes in your comfort levels with breathing since commencing the oxygen or nitric oxide gas:

| Marked deterioration  (-2) | Slight deterioration  (-1) | No change (0) | Slight improvement  (+1) | Marked improvement  (+2) |  |
| --- | --- | --- | --- | --- | --- |
|  |  |  |  |  | |

**Q4**. Please rate your current level of anxiety using the scale^52^ below.

| Not at all anxious  (1) | A little anxious  (2) | Moderately anxious (3) | Very anxious  (4) | Extremely anxious  (5) |  |
| --- | --- | --- | --- | --- | --- |
|  |  |  |  |  | |

**Appendix 2**

The AKIN classification/staging system of acute kidney injury ^48^ ^a^

| Stage | SCr | UO |
| --- | --- | --- |
| 1 | ↑ SCr ≥26.5 μmol/L (≥0.3 mg/dL) or ↑SCr ≥150 a 200% (1.5 a 2×) | <0.5 mL/kg/h (>6 h) |
| 2 | ↑ SCr >200 a 300% (>2 a 3×) | <0.5 mL/kg/h (>12 h) |
| 3^b^ | ↑ SCr >300% (>3×) or if baseline SCr ≥353.6 μmol/L (≥4 mg/dL) ↑SCr ≥44.2 μmol/L (≥0.5 mg/dL) | <0.3 mL/kg/h (24 h) oranuria (12 h) |

^a^SCr, serum creatinine; UO, urine output.

^b^Stage 3 also includes patients requiring RRT independent of the stage (defined by SCr and/or UO) they are in at the moment they initiate RRT.
